# Tryptophan Metabolism, Regulatory T Cells, and Inflammatory Bowel Disease: A Mini Review

**DOI:** 10.1155/2020/9706140

**Published:** 2020-06-12

**Authors:** Xueyan Ding, Peng Bin, Wenwen Wu, Yajie Chang, Guoqiang Zhu

**Affiliations:** ^1^College of Veterinary Medicine, Yangzhou University, Yangzhou 225009, China; ^2^Jiangsu Co-Innovation Center for Important Animal Infectious Diseases and Zoonoses, Yangzhou 225009, China; ^3^Joint International Research Laboratory of Agriculture and Agri-Product Safety, the Ministry of Education of China, Yangzhou University, Yangzhou, Jiangsu 225009, China

## Abstract

Inflammatory bowel disease (IBD) is a chronic inflammatory disorder of the gastrointestinal tract resulting from the homeostasis imbalance of intestinal microenvironment, immune dysfunction, environmental and genetic factors, and so on. This disease is associated with multiple immune cells including regulatory T cells (Tregs). Tregs are a subset of T cells regulating the function of various immune cells to induce immune tolerance and maintain intestinal immune homeostasis. Tregs are correlated with the initiation and progression of IBD; therefore, strategies that affect the differentiation and function of Tregs may be promising for the prevention of IBD-associated pathology. It is worth noting that tryptophan (Trp) metabolism is effective in inducing the differentiation of Tregs through microbiota-mediated degradation and kynurenine pathway (KP), which is important for maintaining the function of Tregs. Interestingly, patients with IBD show Trp metabolism disorder in the pathological process, including changes in the concentrations of Trp and its metabolites and alteration in the activities of related catalytic enzymes. Thus, manipulation of Treg differentiation through Trp metabolism may provide a potential target for prevention of IBD. The purpose of this review is to highlight the relationship between Trp metabolism and Treg differentiation and the role of this interaction in the pathogenesis of IBD.

## 1. Introduction

Inflammatory bowel disease (IBD) is an autoimmune disease with high incidence and unclear etiology, mainly including ulcerative colitis (UC), Crohn's disease (CD), and indeterminate colitis (IC) [1]. UC is an ulcerative bowel disease that only occurs in the colon with a slow and occult onset, and usually, it has a tendency to recur. CD is a chronic, proliferative, and transmural inflammatory disease that can invade any part of the gastrointestinal tract in a discontinuous manner [2]. IBD can seriously lower the quality of lives of patients and significantly increase the risk to colon cancer that result from the proneoplastic effects of chronic intestinal inflammation [3]. A variety of factors, such as genetic, environmental, and microbial factors, are all known to be responsible for the occurrence of IBD [4, 5]. In addition, multiple immune cells like macrophages, dendritic cells (DCs), and lymphoid cells play important roles in the development of IBD, and the turbulence in the differentiation and function of certain T lymphocytes [e.g., regulatory T cells (Tregs)] could contribute to the pathogenesis of IBD [6, 7]. Hence, the thorough understanding of precise regulation of Tregs may be helpful to perceive IBD-related pathology.

Usually, the generation, differentiation, and function of Tregs are significantly affected by the availability of amino acids in the local microenvironment. Depletion of certain essential amino acids from the local milieu results in the generation of Tregs [8–10]. For example, low concentrations of Trp inhibit T cell growth but enhance Treg production through mTOR-dependent mechanisms [11]. In the gastrointestinal tract, Trp undergoes several different metabolic pathways, and Trp metabolism can influence the differentiation and function of Tregs. Trp catabolism is a tolerogenic effector system in Treg function, and its modulation is thought to function as a general mechanism of action of Tregs that express T-lymphocyte antigen-4 (CTLA-4) [12]. In addition, Trp starvation and Trp catabolites could induce the generation of a regulatory phenotype in naive CD4^+^ T cells, and previous studies indicated that there is a close relationship between indoleamine 2,3-dioxygenase (IDO) activity and the occurrence of Tregs [12–14]. Notably, Trp metabolism disorder is also associated with the development and progression of IBD [15–17]. For example, the decreased Trp concentration and increased kynurenine (Kyn) concentration are observed in the IBD patients, and the activity of IDO is also altered as well [18–23]. Thus, regulation of Tregs through altering Trp metabolism may provide potential targets for prevention of IBD.

Herein, we provide an in-depth review highlighting the understanding of the regulatory roles of Trp metabolism in Treg differentiation and discuss the availability of manipulating Trp metabolism to Tregs, which further prevent or ameliorate IBD.

## 2. Tregs and IBD

### 2.1. The Mechanism of Action of Tregs in IBD

In normal intestinal mucosa, effector cells and Tregs are in a state of dynamic equilibrium. Tregs play an important role in maintaining intestinal homeostasis and can significantly suppress immune responses to maintain autoimmune tolerance and immune stability through multiple ways, such as cell-cell contact or cytokine-dependent mechanism [24].

#### 2.1.1. Cell-Cell Contact Mechanism

CD4^+^CD25^+^ Tregs can constitutively express inhibitory regulatory molecules such as cytotoxic CTLA-4, transforming growth factor *β* (TGF-*β*) and glucocorticoid-induced TNF receptor (GITR), which can bind to the corresponding receptors and transmit inhibition signals to prevent excessive activation of target immune cells [25]. The binding is capable of inhibiting the expression of IL-2R*α* chain and reduce the reactivity of target cells to IL-2, thereby inhibiting the proliferation of effector T cells (Teffs). A variety of ligand-receptors including costimulatory molecules such as CTLA-4, GITR, OX40 (CD134), and lymphocyte activation gene 3 (LAG-3) are involved in this process [26–29]. Thus, costimulatory molecule receptors play a significant role in the activation process of Tregs. Studying the mechanisms of their abnormal expression and on how to regulate the signaling pathways may bring new light for a deeper understanding of the mechanism of action of Tregs. In addition, Tregs also express programmed death receptors and ligands, which stabilize the relationship between Tregs and antigen presenting cells (APCs) while promoting the differentiation of inducible regulatory T cells (iTregs) [30]. Moreover, Tregs can downregulate the expression levels of costimulatory molecules CD80 and CD86 on DCs and affect the function of DCs, thereby achieving immunosuppressive effects [31].

#### 2.1.2. Cytokine-Dependent Mechanism

Tregs can achieve their functions by releasing inhibitory cytokines such as interleukin-10 (IL-10), TGF-*β*, and interleukin-35 (IL-35). High mRNA expression of IL-10 and TGF-*β* was found in the CD4^+^CD25^+^ Tregs *in vitro*, and CD4^+^CD25^+^ Tregs can directly secrete IL-10 and TGF-*β* under appropriate stimulation [32]. In the CD4^+^CD45RB^high^ T-induced IBD model, TGF-*β* and IL-10 play an important role in the protective effect of Tregs on IBD. CD4^+^CD25^+^ Tregs isolated from TGF-*β* knockout mice or CD4^+^CD45RB^low^ T cells derived from IL-10 knockout mice lost their anti-IBD function [33, 34]. IL-35 is a heterodimeric cytokine comprising Epstein-Barr virus-induced gene 3 (Ebi3) and IL-12 alpha (IL-12*α*) chain, which was expressed in Foxp3^+^ Tregs, and Tregs lacking Ebi3 or IL-12*α* lost their inhibition in the T cell metastatic colitis model. Exogenous IL-35 inhibits T cell proliferation, and the vector encoding IL-35 achieves *in vitro* inhibitory activity by retroviral transduction into Teffs [35]. More potential mechanisms of action of Tregs in IBD have not been established. Nevertheless, strategies that induce the generation of a regulatory phenotype may be a treatment option in preventing or improving the pathological process of IBD.

### 2.2. Tregs Are Associated with the Development and Progression of IBD

**Figure 1:**
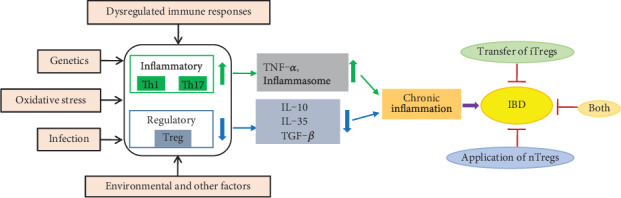
The occurrence of IBD and its relationship with Tregs. The pathophysiology of IBD is multifactorial and not completely understood, but genetic components, dysregulated immune responses, oxidative stress, and inflammatory mediators are known to be involved. Tregs are related to the occurrence and development of IBD, and IBD can be cured or alleviated by inducing the generation of Tregs or direct administration of Tregs. Treg: regulatory T cell; TNF-*α*: tumor necrosis factor *α*; TNF-*β*: tumor necrosis factor *β*; iTregs: inducible regulatory T cells; nTregs: natural regulatory T cells; IBD: inflammatory bowel disease.

Available evidence suggests that Tregs play an important role in the development and immune regulation of IBD (Figure 1). It has been demonstrated that Tregs maintain intestinal homeostasis and reduce tissue damage during the progression of IBD by inhibiting the responsiveness of immune cells [36, 37]. Changes in the number, phenotype, and inhibitory function of Tregs may contribute to the pathogenesis of IBD. For example, Tregs from mice deficient in cytotoxic CTLA-4, IL-35, IL-10, or LAG-3 are unable to effectively suppress T cell proliferation and fail to prevent chronic T cell-mediated colitis *in vivo* [28, 29, 35, 38]. In addition, Tregs in the inflamed mucosa or periphery blood of patients with IBD or animal models are considerably different [39, 40]. For example, Maul et al. found that CD4^+^CD25^+^ Tregs were reduced in peripheral blood during the active phase of IBD, while the frequency of Tregs at the mucosal level was higher than healthy controls [41]. Moreover, the frequency of Foxp3^+^ Tregs was found to be significantly lower in patients with active IBD [42]. In addition, Wang et al. [6] suggest that insufficient Tregs in peripheral blood may be associated with the recurrence of IBD. However, there are still reports that Tregs fail to exert the inhibition function in the context of IBD [43, 44], which might be explained by the individual differences of patients. Therefore, understanding Tregs in IBD can be helpful in monitoring the cellular immune status of IBD patients and opening up new immunotherapeutic approaches for the treatment of IBD.

Mechanistically, Tregs could be considered as therapeutic targets for controlling IBD (Figure 1). Fortunately, many cases of IBD have been successfully cured or alleviated by manipulating Tregs in animal models or patients [45–56]. For example, Treg transfer is sufficient to alleviate experimental colitis including IBD, and tTregs and iTregs can work together [57–60]. Tregs and IL-10 producing Tr1 cells have the potential to prevent or cure colitis, which is supported by a favourable safety profile in phase I clinical trials [61]. However, it should not be overlooked that Treg-based therapies may be attached by some adverse reactions. For example, excessive Treg activity may simultaneously weaken the protective immunity against pathogens and tumors, which could be reduced by controlling the antigen specificity of Tregs [62]. In addition, the phenotype of the original population and culture conditions are also critical for achieving maximum purity of therapeutic Tregs and ensuring phenotypic stability [63, 64]. Therefore, before developing new strategies to improve Treg function, it is very important to study the detailed mechanism of how Tregs function to limit potential negative side effects. It would be meaningful to explore whether it can be more effective when Treg-based therapy is combined with other therapies.

## 3. Trp Metabolism in the Differentiation and Function of Tregs

### 3.1. Trp Metabolism in the Gut

Trp is ubiquitous in many foods and has important physiological functions. Once in the gastrointestinal tract, Trp enters several different metabolic pathways by host or intestinal microbiota [65]. We mainly focus on microbial-mediated degradation, KP, and serotonin pathway. About 4-6% of Trp undergoes microbial degradation, by which intestinal microbes directly convert Trp into several molecules, including indoles and its derivatives [66]. Notably, KP is the major route for Trp catabolism which is mediated by the rate-limiting enzyme IDO1. KP can produce Kyn and its downstream products such as quinolinic acid (QA), niacin, nicotinamide adenine dinucleotide (NAD), and kynurenic acid (KA) [67, 68]. KP metabolites are associated with many biological processes involved in neurotransmission, inflammation, and immune responses. In addition to KP, approximately 1-2% of the dietary Trp is converted to serotonin mediated by tryptophan hydroxylase 1 (TpH1) [69]. There is evidence of the importance of serotonin in regulating gastrointestinal function [70, 71]. Collectively, Trp and its metabolites are essential for the development and maintenance of human and animal health, and all these metabolic pathways work together to maintain the homeostasis.

### 3.2. Trp Promotes Treg Differentiation through Microbiota-Mediated Degradation

**Figure 2:**
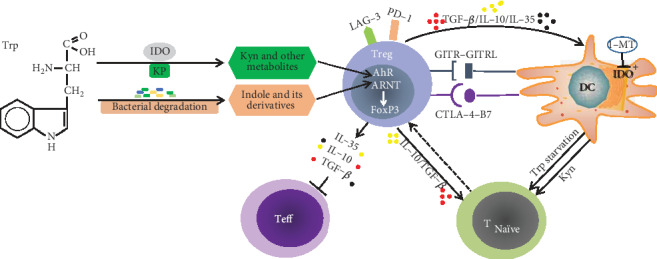
The schematic representation of Trp metabolism and its influence on Tregs. Trp metabolism produces AhR ligands through KP and microbial-mediated degradation, which affects the generation of Tregs. The relationship between IDO and Tregs is bidirectional, because they can regulate each other via DCs. CTLA-4, GITR, IL-10, IL-35, TGF-*β*, and IFN-*γ* are main components of the regulatory responses. Trp: tryptophan; IDO: indoleamine 2, 3-dioxygenase; KP: kynurenine pathway; Kyn: kynurenine; Treg: regulatory T cell; AhR: aryl hydrocarbon receptor; ARNT: aryl hydrocarbon receptor nuclear translocator; FoxP3: forkhead box P3; IL-35: interleukin-35; IL-10: interleukin-10; TGF-*β*: transforming growth factor beta; GITR: glucocorticoid-induced TNF receptor; CTLA-4: cytotoxic T-lymphocyte antigen-4; DC: dendritic cell; 1-MT: 1-Methyl-tryptophan.

Intestinal microorganisms can directly catabolize Trp into indoles and its derivatives, which play an important role in regulating intestinal immune tolerance [72]. Most indoles and its derivatives, such as indole-3-aldehyde (IAld), indole-3-acid-acetic (IAA), indole-3-propionic acid (IPA), indole-3-acetaldehyde (IAAld), and indoleacrylic acid (IA), are the ligands of aryl hydrocarbon receptor (AhR) (Figure 2).

AhR is a ligand-activated transcription factor that is widely found in immune cells and intestinal epithelial cells, and it is sensitive to certain environmental chemicals and plays an important role in the immune response. Previous work demonstrated the importance of AhR in the differentiation and function of Tregs and Teffs by controlling the production of IL-10 and IL-22 [73–77]. Indole and its derivatives infiltrate into intestinal epithelial cells and deposit in the host circulatory system, which could be recognized by immune cells and then activated AhR signaling pathway. It has been well demonstrated that AhR signaling can induce the proliferation of CD4^+^CD25^+^Foxp3^+^ Tregs (Figure 2), which play an indispensable role in adaptive immune tolerance, such as inhibiting the immune function of activated T cells [78–80]. Collectively and mechanistically indole and its derivatives derived from Trp regulate the differentiation of Tregs through AhR-ligand-Treg axis, thereby affecting the function of Tregs [81–90].

### 3.3. Trp Promotes Treg Differentiation through KP

KP is the main pathway of Trp catabolism, through which Kyn and other metabolites are produced, such as KA, anthranilic acid (AA), 3-hydroxykynurenine (3-HK), xanthurenic acid (XA), and QA [91–93]. Some KP metabolites bind to AhR to induce FoxP3 expression and promote the generation and differentiation of FoxP3^+^ Tregs [75, 94–97] (Figure 2). In addition, 3-HK and the downstream product pyridine-2-3-dioxoic acid can trigger the activity of Tregs. This is consistent with the long-term synergistic effect of Trp deficiency, and high Kyn induced the transformation of naive CD4^+^ T cells into Tregs [12].

Since IDO is the main enzyme that catalyzes Trp to produce Kyn and other metabolites, the level of IDO expression is important for KP [98, 99]. IDO is expressed in APCs, and its immunoregulatory function is mainly achieved by DCs. IDO suppresses CD4^+^ T cell function by inhibiting cell proliferation, inducing apoptosis and promoting cell differentiation into Tregs. This is achieved by degrading Trp in the microenvironment where immune responses occur [100, 101]. Francesca et al. found that there was a positive regulatory loop by which Tregs expand their own population through the IDO mechanism. In contrast, the activity of IDO enzyme can be inhibited by 1-Methyl-tryptophan (1-MT) [102, 103]. Therefore, manipulating the activity of IDO or the application of synthetic Kyn could provide an idea for the therapeutic agents of IBD [104]. Later, it was discovered that the relationship between IDO and Tregs was bidirectional [96] (Figure 2). IDO can induce the production of Tregs, and the increase of Tregs can in turn induce the expression of IDO [105]. Given the complex relationship between IDO and Tregs, combining IDO blockade with other immunotherapies may be beneficial to overcome the shortcomings of immune counterregulation.

Likewise, serotonin has been reported to be involved in the pathogenesis of experimental colitis [106, 107]. Therefore, Trp metabolism in the gut is a target that can be considered, such as using either molecules targeting a specific pathway or exploiting bacteria affecting Trp metabolism as probiotics. However, the complicated interactions between microbes and hosts need to be elucidated to achieve better therapeutic effects. Moreover, the metabolic pathways influencing Treg differentiation and function are amenable for modulation in therapeutic settings, thus providing the clinician with potentially valuable tools in the fight against immune-mediated diseases. At the same time, the deviation between diseases and models requires further investigation to refine targets and therapeutic interventions.

## 4. Modulation of Trp Metabolism in Tregs for IBD

**Figure 3:**
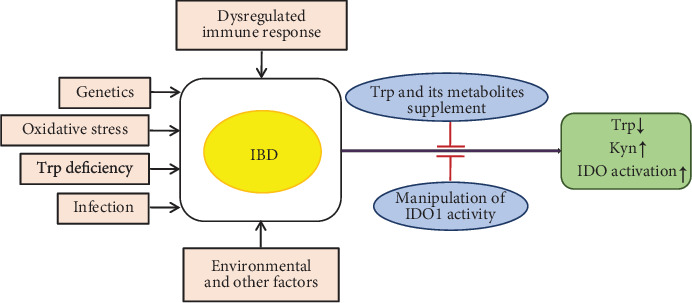
Effects of Trp and its metabolism on the etiology of IBD. Trp deficiency could contribute to the development of IBD, and patients with IBD have lower Trp levels, higher Kyn levels, and elevated IDO expression. Trp: tryptophan; IBD: inflammatory bowel disease; IDO1: indoleamine 2,3-dioxygenase-1; Kyn: kynurenine.

Because Trp metabolism has an important effect on Treg differentiation and function, measures that target Trp metabolism may reduce the severity of IBD, but the possibility deserves further exploration. However, most investigations about effects of Trp metabolism on Treg differentiation were conducted *in vitro* with mouse Tregs; it is not known if Trp metabolism has similar effects in human Treg differentiation *in vitro* or *in vivo*. Indeed, accumulating evidence suggests that Trp promotes intestinal integrity and function, and its metabolism has an important effect on spontaneous and induced IBD models [108–111]. Trp and its metabolism show a high correlation with the etiology of IBD (Figure 3). Usually, Trp deficiency could contribute to the development of IBD or aggravate disease activity [17, 112]. Patients with IBD have lower levels of Trp in serum and feces than healthy subjects [18, 19, 113–115]. Moreover, some Trp metabolites and metabolic enzymes are also found to be significantly different in patients and healthy volunteers [20, 116–118]. Increased Kyn and Kyn/Trp ratios were observed in IBD patients indicating that Trp metabolism along the KP is increased in active IBD [20, 21]. In addition, consumption of Trp metabolites in the intestinal tract may affect the severity of IBD. For example, the concentration of the AhR agonist IAA in feces of IBD patients was significantly reduced [19]. Also, the content of IPA in circulating serum from patients with active colitis was selectively diminished compared to healthy subjects [119, 120]. However, IDO1 levels in the intestine are higher in patients with IBD, although the role of IDO1 in colitis is somewhat controversial [22, 23]. Besides, the content of serotonin in the intestine has changed dramatically in human IBD and animal models of colitis, which suggests that serotonin plays an important role in the occurrence and development of intestinal inflammation [107, 121, 122].

Conversely, dietary supplementation with Trp and Trp metabolites could alleviate symptoms such as weight loss, fecal hemorrhage, and colonic structural damage in experimental mouse colitis (Figure 3) [72, 106]. The protective effect of Trp administration on IBD may be achieved by reducing proinflammatory cytokines and activating apoptosis initiators, while another anticolitis mechanism may be antioxidative or nitration stress [106, 123]. For example, dietary supplementation of 0.5% Trp inhibited colonic inflammatory symptoms and proinflammatory cytokine secretion in mice by activating AhR [124]. Mice or piglets fed a Trp-supplemented diet had reduced inflammation and decreased severity of dextran sodium sulfate- (DSS-) induced colitis [112, 123, 125], whereas mice fed a low­Trp diet became susceptible to chemically induced inflammation. In addition, the administration of Trp metabolites, such as Kyn, indole, and IPA, were observed to ameliorate colonic inflammation in mice [119, 120, 126]. Simultaneously, manipulation of IDO1 activity has great potential as treatment for IBD [127]. Moreover, indirect manipulation of the gut microbiota affecting Trp metabolism could be considered to develop new therapeutic drugs that target IBD individuals. For example, the use of *Lactobacillus* (a kind of bacterium that degrades Trp into AhR agonists) lightened the severity of colitis in mice, and probiotics can serve as a supportive therapy for patients with intestinal disorders [125]. Collectively, Trp and its metabolites can be used as biomarkers and promising targets for the treatment of IBD, but further investigation is necessary to validate the effectiveness and feasibility [17, 128]. Therefore, the levels of Trp and its metabolites in patients with IBD need to be analyzed to assess their impact on the progression of IBD.

Collectively, patients with IBD have lower Trp levels, higher Kyn levels, and elevated IDO expression. This is positively correlated with reduced Tregs in IBD. Thus, manipulation of Treg differentiation through these metabolites may be a promising strategy for the treatment of IBD.

## 5. Conclusions and Future Perspectives

**Figure 4:**
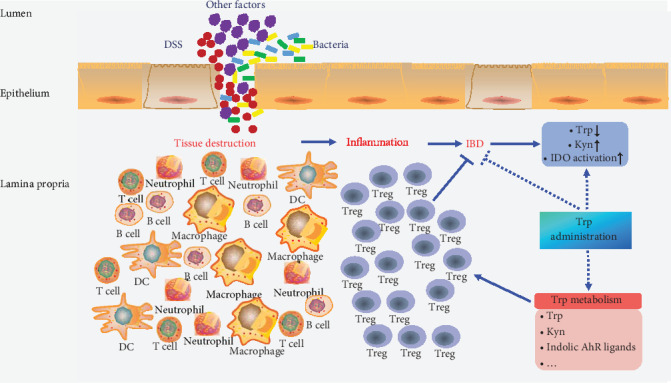
Overview of the relationship among Trp metabolism, Tregs, and IBD. DSS: dextran sodium sulfate; Treg: regulatory T cell; IBD: inflammatory bowel disease; Trp: tryptophan; Kyn: kynurenine; IDO: indoleamine 2,3-dioxygenase; AhR: aryl hydrocarbon receptor.

In summary, Tregs are associated with the development of IBD and strategies to manipulate Treg differentiation by Trp metabolism may lead to new therapeutic approaches for the treatment of IBD (Figure 4). Therefore, it is important for researchers to elucidate the exact regulatory mechanism of Trp metabolism in Tregs during the development of IBD. Fortunately, Trp and its metabolites are known to be beneficial for IBD patients and related animal models, although it is unclear whether they regulate the progression of IBD by precisely affecting the differentiation and function of Tregs. Considering that other metabolic pathways also regulate the proliferation and function of Tregs (such as the CD39-CD73-adenosine pathway), combining Trp metabolism with other metabolic pathways will be a better strategy for preventing IBD-related pathologies. At the same time, the manipulation of metabolic pathways in Tregs can be combined with traditional drugs that affect Treg function to achieve better preventive and therapeutic effects. But at present, very satisfactory results have not been achieved in the treatment of IBD. Therefore, an in-depth study of the role of immune cells and amino acid metabolism in IBD will provide a more meaningful basis for the early diagnosis, effective treatment, and progression evaluation of IBD, which will be a serious challenge in the medical field.
